# Characterization and Topical Study of Aloe Vera Hydrogel on Wound-Healing Process

**DOI:** 10.3390/polym13223958

**Published:** 2021-11-16

**Authors:** Karen Zulema Meza-Valle, Rosa Alicia Saucedo-Acuña, Karla Lizzette Tovar-Carrillo, Juan Carlos Cuevas-González, Erasto Armando Zaragoza-Contreras, Juana Melgoza-Lozano

**Affiliations:** 1 Institute of Biomedical Sciences, Autonomous University of Ciudad Juarez, Av. Benjamín Franklin No. 4650, Zona Pronaf Condominio La Plata, Ciudad Juárez 32310, Mexico; mvz.karenmeza@gmail.com (K.Z.M.-V.); karla.tovar@uacj.mx (K.L.T.-C.); cuevas_gonzalez@hotmail.com (J.C.C.-G.); 2 Centro de Investigación en Materiales Avanzados, S.C. Miguel de Cervantes No. 120, Complejo Industrial Chihuahua, Chihuahua 31136, Mexico; armando.zaragoza@cimav.edu.mx; 3 Derseg Co., Abasolo No. 205 Zona Centro, Apaseo el Grande 38160, Mexico; juanita.melgoza@gmail.com

**Keywords:** Aloe vera, hydrogel, support matrix, skin regeneration, wound healing

## Abstract

Wound healing is fundamental to restore the tissue integrity. A topical study of the influence of Aloe vera hydrogel, formulated with 1,2-propanediol (propanediol) and triethanolamine (TEA), on the skin wound-healing process was investigated in female Wistar rats. FTIR spectroscopy confirms the presence of carboxylic acid and methyl ester carboxylate groups related with important compounds that confer the hydrogel a good interaction with proteins and growth factors. SEM images show a microstructure and micro-roughness that promote a good adhesion to the wound. Therefore, the swelling kinetics and the contact angle response contribute to the understanding of the in vivo results of the animal test. The results indicated that the Aloe vera hydrogel, prepared with propanediol and TEA, together with its superficial characteristics, improve its rapid penetration without drying out the treated tissue. This produced a positive influence on inflammation, angiogenesis, and wound contraction, reducing 29% the total healing time, reaching the total closure of the wound in 15 days.

## 1. Introduction

Polymeric biomaterials combined with natural extracts are an important line of research in the field of tissue engineering as regeneration matrices or scaffolds [1,2]. Among scaffolds, natural hydrogels stand out because of their high water content, biocompatibility, and biodegradation. A hydrogel is a cross-linked hydrophilic polymer that exhibits a high water content similar to tissue, which provides permeability to nutrients and adequate adherence to surrounding tissue [2,3,4].

In 2020, the global Aloe vera hydrogel market was valued at 649.41 million USD, with the pharmaceutical industry being the main end-user with 61% of consumption [5]. Aloe vera extracts are widely used to treat skin lesions due to their healing, anti-inflammatory, analgesic, vasodilatory, and antimicrobial properties [6,7,8,9,10]. The complex composition of the hydrogel includes about 75 potential compounds, including minerals and phenolics [5,11,12]. Aloe-emodin, aloesin, aloin, and acemannan are some of the most studied compounds in Aloe vera, where acemannan is known to induce tissue repair [13,14,15,16,17,18]. In addition, these polysaccharides have been explored as a functional source for biomedical and pharmaceutical materials due to their natural gelling mechanism, high availability, and nontoxic nature [19,20,21,22].

Some clinical and experimental evidence suggested the usefulness of Aloe vera in wound care and wound healing for skin moisturization, promoting wound healing, thermal skin injury, and inflammatory skin pathologies [17,23,24,25,26,27,28]. Therefore, in vitro/in vivo trials using Aloe vera gel, as a scaffold or support matrix for tissue engineering, have received considerable attention either alone or in combination with conventional drugs or natural extracts [5,11,29,30,31,32,33,34,35,36,37,38,39,40,41,42,43,44,45]. For instance, the use of fresh Aloe vera gel for 10 days to treat breast soreness and irritation in lactating women produced significant wound healing [43]. In addition, a commercial Aloe vera gel formulated with allantoin, bisabolol, and vitamin E was used to treat patients with vitiligo after phototherapy sessions. A significant reduction of burning sensation and high reduction in scores were observed after two and eight weeks, respectively [38]. Furthermore, Aloe vera powder was used to prepare a cream to treat vaginal atrophy for menopausal women. The treatment revealed a significant improvement in the cytological and clinical indices of vaginal atrophy, demonstrating that Aloe vera can be used safely [40]. Frequently, Aloe vera powder is obtained by freeze drying [34].

Differences in the composition of Aloe vera hydrogels are related to geographical and seasonal variations and the elaboration process [5,12]. In some studies, the selected leaves were rinsed with water or even with a mild chlorine solution as a disinfectant [10,35]. After the hydrogel extraction, for the sterilization, some works selected UV radiation [36], pasteurization, or a cooling process [5] during the homogenization of the hydrogel. Finally, the hydrogel needs to be stabilized with different compounds to prevent separation in products or water loss, maintain a desired pH, or achieve other physical properties such as viscosity or flexibility [29].

Derseg Co. elaborates a cosmetic product based on a hydrogel of Aloe vera denominated Restauder^®^. This hydrogel was designed to treat skin cosmetic affections such as acne scars. Restauder^®^ contains, among other additives, parabens as antibacterials [46,47], glycerin as plastificant [29], carbomer to control the consistency [48], propanediol to confer a non-sticky feel, and triethanolamine to stabilize the product. These compounds do not contribute to the wound-healing process, but they are widely used in cosmetics and personal care products. Propanediol is a natural alternative to propylene glycol as a common solvent, humectant, and emollient. It allows the formulas to flow well and makes them easier to use on the skin [49,50,51]. On the other hand, TEA is a common and safe stabilizer and pH adjuster in skincare cosmetics [52,53].

The effective results in 4 days of skin wound treatment using Restauder^®^ aroused interest in promoting its use in the treatment of deep dermis wounds as a pharmaceutical proposal [54]. Consequently, this work aimed to evaluate this commercial hydrogel and demonstrate that the presence of the specific additives, propanediol and triethanolamine, do not exert any negative effect on wound healing. The effectiveness of the product as an accelerator of cutaneous wound healing was demonstrated in an in vivo study as part of the pharmaceutical validation of Restauder^®^ for clinical use.

## 2. Materials and Methods

### 2.1. Restauder^®^ Hydrogel

Restauder^®^ hydrogel was donated by Derseg Co. (Apaseo El Grande, Guanajuato, Mexico). Three Restauder^®^ products from three different manufacturing batches were used during the analysis. The three batches were manufactured six months before the realization of this study.

### 2.2. Characterization of the Hydrogel

For characterization, the hydrogel was dried to obtain xerogels. This is necessary to avoid water interference with FTIR and SEM analysis and also to perform the swelling study. Xerogels are prepared by drying hydrogels slowly at 35 °C over 4 weeks. This has the advantage that xerogels retain their porosity (at least in part) after the drying process [55].

For the water-swelling assay, three xerogel samples were cut into 3 pieces of 5 mm × 5 mm. A swelling study was conducted gravimetrically at room temperature using distilled water [7]. Finally, the samples were removed, and the swelling degree (*%S*) was calculated according to Equation (1).

(1) $$\%S=\frac{\left(W_{s}-W_{d}\right)}{W_{d}}\times 100$$

where *W_s_* and *W_d_* stand for the weight of the hydrated and dry samples, respectively [56,57].

Fourier transform infrared spectra were acquired using an Alpha-T FTIR system (Bruker, Billerica, MA, USA) to examine chemical functional groups in dried hydrogel samples in the wavenumber range from 4000 to 400 cm^−1^. Surface morphology and topography of the film were recorded by optical microscopy (AX70, Olympus, Tokyo, Japan) and scanning electron microscopy (JSM-6010Plus, JEOL, Akishima, Japan) under a vacuum of 60 Pa and 15 kV. For the analysis of the sample, no treatment was required, only low vacuum. Finally, a contact angle meter (FTA-32, First Ten Ånstroms, Portsmouth, VA, USA) was used to calculate the angle of the samples by the sessile drop method.

### 2.3. In Vivo Assay

Female Wistar rats with a weight of 250 g were used for the assay, which were previously approved by the Animal Ethics Committee of the Autonomous University of Ciudad Juarez (approval number CIBE-2017-1-45). For this, 27 Wistar rats were used, to which a total thickness wound of 2 cm in diameter was made on the back using a scalpel. The specimens were placed in polycarbonate cages for a month at 21 °C and 45% of humidity with an ad libitum diet [58,59]. The rats were randomly distributed into two groups: Group C with 15 animals, where the wounds received no treatment, and Group D with 12 animals, in which the wounds were treated with the Restauder^®^ hydrogel.

Images were taken with a comparative scale at defined times to later make precise measurements with the SolidWorks (Dassault Systèmes, Vélizy-Villacoublay, France) program and calculate the rate of wound contraction to make the comparison between the groups. Likewise, biopsies were taken to assess the quality of the healing tissue between both groups [19].

#### 2.3.1. Surgical Procedure

For surgery, rats were anesthetized using xylazine (10 mg/kg) and tiletamine/zolazepam (30 mg/kg) by the intramuscular route in the gluteal region. For in vivo assay, surgical excision of 2 cm in diameter was made in the dorsal area [60,61]. After the surgery and every 2 h daily, from 8 until 20 h, the area exposed was covered with a layer of hydrogel for the rest of the experiment. Even a scab developed on the wound, and the hydrogel was placed over and on the edge of the wound. The biopsies were performed at five days, varying the lifespan of the rats (0 for only Group C and 4, 8, 15, and 21 days for both groups). Each time, 3 rats were biopsied. In total, 15 rats for Group C and 12 rats for Group D were used.

#### 2.3.2. Macroscopic Analysis

**Figure 1:**
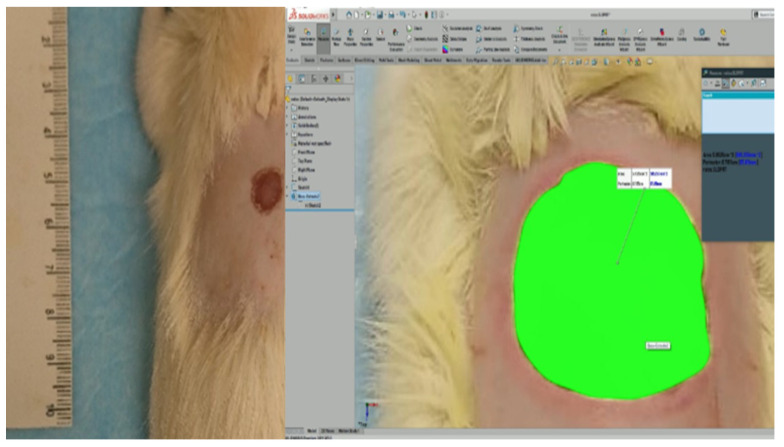
Reference scale (**left**) and the measure of the wound’s area by SolidWorks (**right**).

To measure the wound closure, rats were sedated with isoflurane for 30 s to take a photograph of the wound at 0, 4, 8, 15, and 21 days [19]. For the best precision in the measurement, each photograph was analyzed by SolidWorks (Figure 1), comparing the size of the area at day 0 to calculate the contraction of the wound using Equation (2).

(2) $$\%AD=\frac{\left(AD_{0}-AD_{t}\right)}{AD_{0}}\times 100$$

where *AD*_0_ and *AD_t_* stand for the excision area on day 0 and day “*t*”, respectively. “*t*” represents 4, 8, 15, and 21 days.

#### 2.3.3. Microscopy Study

On days 4, 8, 15, and 21, rats were euthanized by an overdose of anesthesia in three members of each group to take a skin biopsy. Samples were extracted through an elliptical incision with a skin margin of 2 cm around the edges of the wound. The extract was preserved in 10% formalin until histological processing [19]. The slides obtained with the longitudinal sections of the biopsies were evaluated with the parameters described in Table 1.

## 3. Results

### 3.1. FTIR Analysis

**Figure 2:**
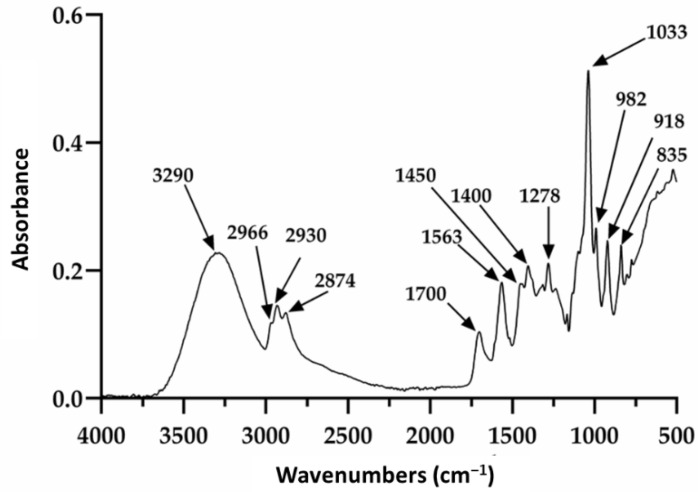
Infrared spectrum of the pure hydrogel.

Restauder^®^ hydrogel, as mentioned elsewhere in the manuscript, is an Aloe vera gel formulated with various additives. Of these, propanediol and triethanolamine are especially observed in this study, since although they do not contribute a therapeutic function to the gel, it is expected that they do not affect its repairing properties. The Aloe vera gel itself consists of a large number of components; therefore, the signals observed in Figure 2 will be discussed based on the reported literature, mainly taking into account the signals for the gel, propanediol and triethanolamine. The absorption at 3290 cm^−1^ is attributed to the presence of the various OH groups contained in the polysaccharide structure of the gel, propanediol and TEA [62,63]. The peaks at 2930 and 2874 cm^−1^ were attributed to the symmetric and asymmetric stretching of the aliphatic C–H bonds of the methylene groups (CH_2_), which are also present in the product components. The signals at 1700 and 1563 cm^−1^ were associated with the stretching of the carbonyl group (C=O) and the carboxylate group (COO^−^), respectively [62]. Additionally, the signals at 1278 and 1235 cm^−1^ have been ascribed to the bending of the glycosidic bond and stretching of the acetyl groups. Finally, the pronounced peak at 1033 cm^−1^ is related to the C–C–O(H) bond of polysaccharides present. It should be noted that this signal is also reported for these same groups in TEA and propanediol. Furthermore, the signals at 2966 and 1450 cm^−1^ are reported for the tension of the aliphatic C–H bonds and are present in triethanolamine and propanediol. On the other hand, the absorptions at 982, 918, and 835 cm^−1^ seem to be mainly related to the presence of propanediol and correspond to stretching of the C–H bond in methyl and methylenes [62,63].

### 3.2. Contact Angle

**Figure 3:**
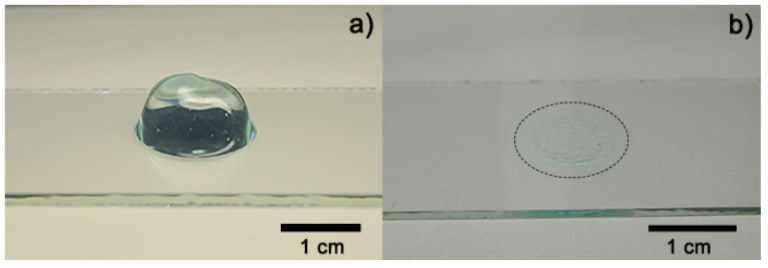
Optical images of Aloe vera hydrogel: (**a**) sample before drying process and (**b**) dried sample (xerogel).

**Figure 4:**
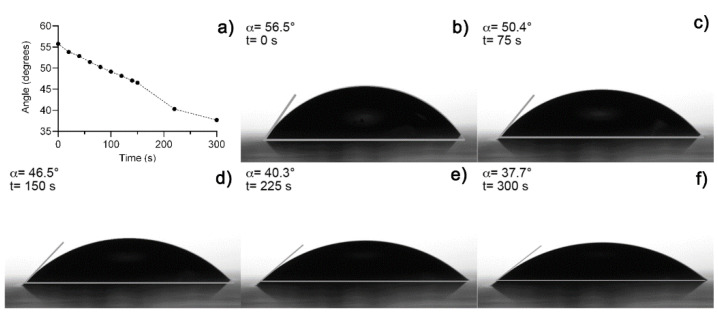
(**a**) Contact angle measurement over time, (**b**) initial measure at *t* = 0 s, (**c**) at 75 s, (**d**) 150 s, (**e**) at 225 s, and (**f**) at 300 s.

Figure 3a shows an image of fresh hydrogel before the drying process (Figure 3b). The hydrogel collapsed after 5 h absorbing 3 mL of distilled water, obtaining a maximum swelling of 12,693% and a recovery of water expected because fresh Aloe vera hydrogels are 98–99% water [12,19]. A media of 43.11° of contact angle measurements was obtained from fresh hydrogel samples. Figure 4 shows the contact angle measurement over time after the collapse of the xerogel sample. Both results were expected because of the presence of functional groups, which were predominantly polar in the hydrogel [10,19].

### 3.3. Surface Analysis

**Figure 5:**
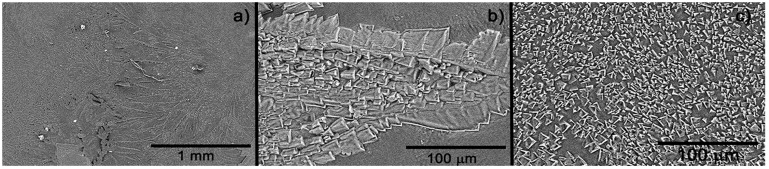
SEM image of the hydrogel dried (xerogel) at various magnifications: (**a**) longitudinal image of the surface, (**b**) approach to dendritic structure, and (**c**) approach to continuous crystalline structure.

Figure 5 portraits SEM images of the dehydrated hydrogel. As noted, a crystalline structure conforms the deposition. At high magnification (Figure 5a), two types of crystalline structures are observed: one dendritic type and the other continuous. Figure 5b, at higher magnification, shows the crystal structure of the dendritic phase, which is made up of crystals of various sizes and geometries. On the other hand, in the continuous or non-dendritic phase, the crystals have a very homogeneous size, in the order of 2 to 15 μm. It is worth saying that this type of topography and morphology improve the adhesion of the hydrogel with surrounding tissue and porous, which will permit draining the excessive moisture in the wound.

### 3.4. Healing Process

**Figure 6:**
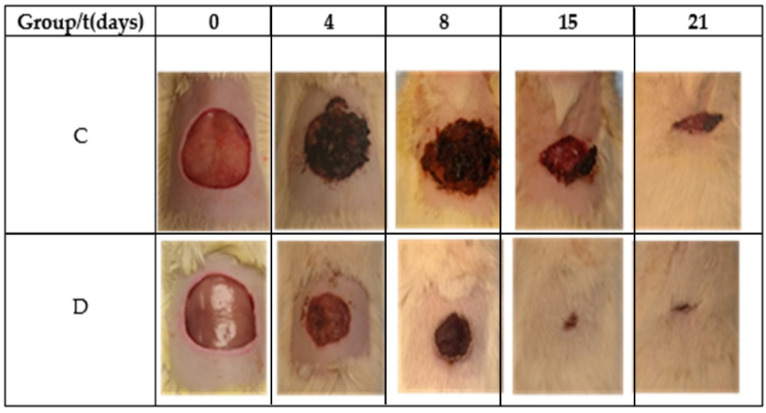
Images of the wound during the healing process.

Table 2 and Figure 6 show the reduction area of the control (Group C) and Restauder^®^ (Group D), where the advantage in the reduction of the area of the wound treated with the hydrogel is clear. For group D on the second day, scabs began to develop, and for day 8, the scab covered all wound.

**Figure 7:**
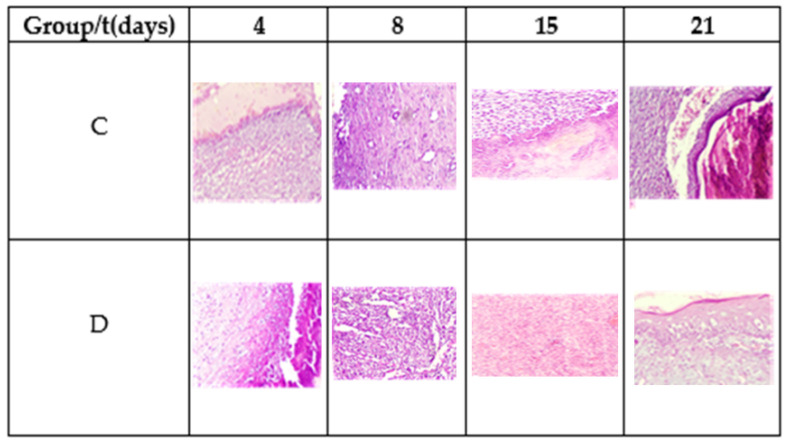
Morphology of the tissue during the healing process. Hematoxylin and eosin stain, 20×.

Table 3 shows the results of microscopic analysis of the wounds at 4, 8, 15, and 21 days of the experiment (Figure 7).

On the fourth postoperative day, a diffuse chronic inflammatory process was observed with a predominance of lymphocytes. In addition, the presence of macrophages and fibroblasts, some angiogenesis, and the absence of epithelium is noted. Fibrin was more abundant in Group D. The observed planimetric analysis results showed a higher wound area reduction of Group D. This might be related to higher fibrin production and distribution.

On the eighth day, diffuse chronic inflammation with a predominance of lymphocytes was less intense in Group D. An abundance of fibrin was also observed in both groups and moderate angiogenesis in Group C and abundant in Group D, as shown in Figure 7. Both groups showed areas of epithelialization in the areas close to the edge of the ulcer. On day 15, moderate inflammation was observed in Group C and less in Group D, as well as the maturation of the fibrin areas and re-epithelialization of the tissues. On day 21, Group C exhibited moderate to scarce inflammatory cells and a completely reconstructed epithelium under a crust in the process of shedding. Meanwhile, the inflammatory cells were scarce to absent in Group D, observing complete healing.

## 4. Discussion

Peaks related to pectin-like rhamnogalacturonan and acemannan were detected in the hydrogel. Both of them are important compounds known to induce tissue repair [13,14,15]. The carboxylic acid and methyl ester carboxylate groups present in these compounds confer to Aloe vera hydrogel a high polarity, which is an advantage for the practical use because it guarantees the adhesion to a wound but with good interaction with proteins and growth factors [6,15,17].

Contact angle measurements and the maximum swelling value show the hydrophilic character of the hydrogel, which is an important property to improve positively the healing process and cell proliferation, improving the clinical appearance and accelerating wound closure [15,24,31,32]. Polar groups will be an advantage in the adsorption of the material, and non-polar groups present in the Aloe vera too will facilitate the bio-integration of the hydrogel with proteins and growth factor into the wound. There has been reported a relationship between the contact angle value and protein adsorption to promote cell adhesion on the material’s surface. It has been shown that contact angle values between 40° and 60° promote cell adhesion and are a more suitable surface for tissue regeneration [31,32,64].

SEM images showed a rough surface, which is a necessary feature to promote better adhesion and interaction of the material in the wound and surrounding tissues [14,19]. Keeping the structure of natural hydrogel is desirable to use the hydrogel as a support matrix to tissue regeneration as a connective tissue does [19]. The results suggested that the wounds treated with the hydrogel show no histological difference, regarding the control wounds in the observed lamellae; however, clinically, the wounds looked better without the presence of abnormal cells. Nevertheless, research using other parameters and biological conditions must be performed.

An in vivo trial determines that the compounds of Aloe vera hydrogel have adequate surface and biocompatible properties as well as good wound-healing promotion from an in vivo test. The data provide sufficient evidence to affirm that the wound contraction speed of the control group is significantly lower than that of the group treated with Restauder^®^ with 95% confidence. On day 4, it was observed that the contraction rate of the wound is higher than the control, showing a positive effect on the healing process. Furthermore, the mathematical equation obtained from SolidWorks^®^ permitted estimating the total closure of the wound of the control group on day 24, while the group treated with the hydrogel completely closed on day 18. It is worth noting that the simulation program showed a 75% reduction in the healing time of the complete healing process in comparison with the control group.

Similar topical studies of the influence of an extract or gel of Aloe vera on the skin wound-healing process in Wistar rats (with a wound made on the back) reported a significantly higher reduction of the scores (almost total wound closure) at 8 days for chitosan–glucan complex fibers reinforced with collagen and embedded with Aloe vera powder [34], 15 days for the pectine–allantoin film [19], and 15 days for insulin-loaded nanoemulsion with fresh Aloe vera gel in animals with induced diabetes [35]. These research findings highlight the importance of the compound present in the hydrogel formulation, regardless of whether it is added fresh or in powder form, since the investigations achieve around 75% complete wound closures in the first 8 days. The results suggest that the Aloe vera hydrogel, a fresh hydrogel enriched with isopropanediol and TEA, of Derseg Co. is competitive for improving the skin wound-healing process, even though Restauder^®^ was used after 6 months of preparation (stored at 18–24 °C), a period after which no decrease in its effectiveness was observed. Furthermore, this work demonstrated that isopropanediol and triethanolamine, present in the formula of Restauder^®^ hydrogel, did not show an adverse effect on the treatment of wounds.

## 5. Conclusions

There is a tendency to prefer the use of fresh Aloe vera hydrogels just extracted from the plant to working with hydrogels or extracts subjected to further processing. This has decreased the lifetime of the active agents or raised costs for refrigeration. This work shows that it is possible to obtain very competitive results with Aloe vera hydrogels processed months in advance and enriched with ingredients widely used in the personal care industry, but which were being neglected in the most recent proposals, such as the case of propanediol and triethanolamine. Consequently, we suggest reconsidering the use of sterilization and stabilization methods that allow a longer useful life of the product to reduce its cost and achieve a greater scope in its distribution. Finally, this research reinforced the fact that the components of Aloe vera, related to pectin-like rhamnogalacturonan and acemannan, make it possible to achieve such a significant difference to improve the skin wound-healing process.

## Figures and Tables

**Table 1:** Evaluation parameters for microscopic analysis [19].

Parameter	Scale			
Inflammation	-	+	++	+++
Angiogenesis	-	+	++	+++
Fibrin	-	+	++	+++
Epithelialization	-	+	++	+++

(-) absent, (+) mild, (++) moderate, (+++) intense.

**Table 2:** Percentage reduction of the wounds during the healing process.

*t* (days)	C (%)	D (%)	Significance
4	18.48 ± 3.12	28.62 ± 6.02	0.041
8	47.49 ± 3.89	56.52 ± 1.53	0.017
15	83.99 ± 2.15	91.60 ± 0.99	0.006
21	93.58 ± 3.70	100	0.048

LSD (Least Significance Difference), Test (*n* = 15, considering the size of day 0 for both groups) for each group test (*p* < 0.05).

**Table 3:** Evaluation of the histological parameters.

Group	*t* (days)	Parameter			
		Inflammation	Fibrin	Angiogenesis	Epithelialization
C	4	+++	++	+	-
	8	+++	+++	++	+
	15	++	+++	+++	++
	21	+	++	++	+++
D	4	+++	+++	+	-
	8	+++-	+++	+++	+
	15	++	++	++	++
	21	-	+	+	+++

(-) absent, (+) mild, (++) moderate, (+++) intense.

## Data Availability

All data obtained from this study can be found in the research archives of the Master Program in ChemistryBiology Science of the Autonomous University of Ciudad Juarez and can be requested through the corresponding author.

## References

[1] Mano J.F., Silva G.A., Azevedo H.S., Malafaya P.B., Sousa R.A., Silva S.S., Boesel L.F., Oliveira J.M., Santos T.C., Marques A.P. (2006). Natural origin biodegradable systems in tissue engineering and regenerative medicine: Present status and some moving trends. J. R. Soc. Interface.

[2] Kesharwani P., Bisht A., Alexander A., Dave V., Sharma S. (2021). Biomedical applications of hydrogels in drug delivery system: An update. J. Drug Deliv. Sci. Technol..

[3] Carrillo K.L.T., Tamayo G., Donohue A., Kobayashi T., Acuna R.A.S. (2015). Obtaining of Hydrogels using PVA and HEC for Adipose Tissue Regeneration. J. Tissue Sci. Eng..

[4] Kong F., Mehwish N., Niu X., Lin M., Rong X., Hu F., Lee B. (2021). Personalized hydrogels for individual health care: Preparation, features, and applications in tissue engineering. Mater. Today Chem..

[5] Maan A.A., Ahmed Z.F.R., Khan M.K.I., Riaz A., Nazir A. (2021). Aloe vera gel, an excellent base material for edible films and coatings. Trends Food Sci. Technol..

[6] Jadhav A.S., Patil O.A., Kadam S.V., Bhutkar M.A. (2020). Review on Aloe Vera is used in Medicinal Plant. Asian J. Res. Pharm. Sci..

[7] Zeng W.M., Parus A., Barnes C.W., Hiro M.E., Robson M.C., Payne W.G. (2020). Aloe vera—Mechanisms of Action, Uses, and Potential Uses in Plastic Surgery and Wound Healing. Surg. Sci..

[8] Anand U., Tudu C.K., Nandy S., Sunita K., Tripathi V., Loake G.J., Dey A., Proćków J. (2021). Ethnodermatological use of medicinal plants in India: From ayurvedic formulations to clinical perspectives—A review. J. Ethnopharmacol..

[9] Anywar G., Tugume P., Kakudidi E.K. (2021). A review of Aloe species used in traditional medicine in East Africa. South Afr. J. Bot..

[10] Farid A., Tawfik A., Elsioufy B., Safwat G. (2021). In vitro and in vivo anti-Cryptosporidium and anti-inflammatory effects of Aloe vera gel in dexamethasone immunosuppressed mice. Int. J. Parasitol. Drugs Drug Resist..

[11] Sánchez M., González-Burgos E., Iglesias I., Gómez-Serranillos M.P. (2020). Pharmacological Update Properties of Aloe Vera and its Major Active Constituents. Molecules.

[12] Nizam N.H.M., Rawi N.F.M., Ramle S.F.M., Aziz A.A., Abdullah C., Rashedi A., Kassim M.H.M. (2021). Physical, thermal, mechanical, antimicrobial and physicochemical properties of starch based film containing aloe vera: A review. J. Mater. Res. Technol..

[13] Chokboribal J., Tachaboonyakiat W., Sangvanich P., Ruangpornvisuti V., Jettanacheawchankit S., Thunyakitpisal P. (2015). Deacetylation affects the physical properties and bioactivity of acemannan, an extracted polysaccharide from Aloe vera. Carbohydr. Polym..

[14] Yagi A., Takeo S. (2003). Anti-inflammatory Constituents, Aloesin and Aloemannan in Aloe Species and Effects of Tanshinon VI in Salvia miltiorrhiza on Heart. Yakugaku Zasshi.

[15] Sierra-García G.D., Castro-Ríos R., González-Horta A., Lara-Arias J., Chávez-Montes A. (2014). Acemannan, an Extracted Polysaccharide from Aloe vera: A Literature Review. Nat. Prod. Commun..

[16] Adlakha K., Koul B., Kumar A. (2021). Value-added products of Aloe species: Panacea to several maladies. S. Afr. J. Bot..

[17] Wahedi H.M., Jeong M., Chae J.K., Gil Do S., Yoon H., Kim S.Y. (2017). Aloesin from Aloe vera accelerates skin wound healing by modulating MAPK/Rho and Smad signaling pathways in vitro and in vivo. Phytomedicine.

[18] Maan A.A., Nazir A., Khan M.K.I., Ahmad T., Zia R., Murid M., Abrar M. (2018). The therapeutic properties and applications of Aloe vera: A review. J. Herb. Med..

[19] Valle K.Z.M., Acuña R.A.S., Arana J.V.R., Lobo N., Rodriguez C., Cuevas-Gonzalez J.C., Tovar-Carrillo K.L. (2020). Natural Film Based on Pectin and Allantoin for Wound Healing: Obtaining, Characterization, and Rat Model. BioMed Res. Int..

[20] Araya-Quintanilla F., Gutiérrez-Espinoza H., Cuyul-Vásquez I., Pavez L. (2021). Effectiveness of aloe vera in patients with type 2 Diabetes Mellitus and pre-diabetes: An overview of systematic reviews. Diabetes Metab. Syndr. Clin. Res. Rev..

[21] Abdollahiyan P., Oroojalian F., Mokhtarzadeh A. (2021). The triad of nanotechnology, cell signalling, and scaffold implantation for the successful repair of damaged organs: An overview on soft-tissue engineering. J. Control. Release.

[22] Majumder R., Das C.K., Mandal M. (2019). Lead bioactive compounds of Aloe vera as potential anticancer agent. Pharmacol. Res..

[23] Liu X., You L., Tarafder S., Zou L., Fang Z., Chen J., Lee C.H., Zhang Q. (2018). Curcumin-releasing chitosan/aloe membrane for skin regeneration. Chem. Eng. J..

[24] Pathalamuthu P., Siddharthan A., Giridev V., Victoria V., Thangam R., Sivasubramanian S., Savariar V., Hemamalini T. (2019). Enhanced performance of Aloe vera incorporated chitosan-polyethylene oxide electrospun wound scaffold produced using novel Spirograph based collector assembly. Int. J. Biol. Macromol..

[25] Rodrigues L.L.O., de Oliveira A.C.L., Tabrez S., Shakil S., Khan M.I., Asghar M.N., Matias B.D., Batista J.M.A.D.S., Rosal M.M., de Lima M.M.D.F. (2018). Mutagenic, antioxidant and wound healing properties of Aloe vera. J. Ethnopharmacol..

[26] Drudi D., Tinto D., Ferranti D., Fiorelli F., Pozzo M.D., Capitani O. (2018). Aloe barbadensis miller versus silver sulfadiazine creams for wound healing by secondary intention in dogs and cats: A randomized controlled study. Res. Veter. Sci..

[27] Mirzaalizadeh B., Sharif M., Daryani A., Ebrahimzadeh M.A., Zargari M., Sarvi S., Mehrzadi S., Rahimi M.T., Mirabediny Z., Golpour M. (2018). Effects of Aloe vera and Eucalyptus methanolic extracts on experimental toxoplasmosis in vitro and in vivo. Exp. Parasitol..

[28] Araújo L.U., Grabe-Guimarães A., Mosqueira V., Carneiro C.M., Silva-Barcellos N.M. (2010). Profile of wound healing process induced by allantoin. Acta Cir. Bras..

[29] Bialik-Wąs K., Pluta K., Malina D., Barczewski M., Malarz K., Mrozek-Wilczkiewicz A. (2020). Advanced SA/PVA-based hydrogel matrices with prolonged release of Aloe vera as promising wound dressings. Mater. Sci. Eng. C.

[30] Costa F.D.C., Vasconcelos E.M., Azevedo V.A.N., Paulino L.R.F.M., Soares M.D., Silva J.R.V., Silva A.W.B., Souza A.L.P. (2021). Aloe vera increases mRNA expression of antioxidant enzymes in cryopreserved bovine ovarian tissue and promotes follicular growth and survival after in vitro culture. Cryobiology.

[31] Ghorbani M., Nezhad-Mokhtari P., Ramazani S. (2020). Aloe vera-loaded nanofibrous scaffold based on Zein/Polycaprolactone/Collagen for wound healing. Int. J. Biol. Macromol..

[32] Yin J., Xu L. (2020). Batch preparation of electrospun polycaprolactone/chitosan/aloe vera blended nanofiber membranes for novel wound dressing. Int. J. Biol. Macromol..

[33] Wang Y., Zhang Y., Lin Z., Huang T., Li W., Gong W., Guo Y., Su J., Wang J., Tu Q. (2021). A green method of preparing a natural and degradable wound dressing containing aloe vera as an active ingredient. Compos. Part B Eng..

[34] Abdel-Mohsen A., Frankova J., Abdel-Rahman R.M., Salem A., Sahffie N., Kubena I., Jancarabh J. (2020). Chitosan-glucan complex hollow fibers reinforced collagen wound dressing embedded with aloe vera. II. Multifunctional properties to promote cutaneous wound healing. Int. J. Pharm..

[35] Chakraborty T., Gupta S., Nair A., Chauhan S., Saini V. (2021). Wound healing potential of insulin-loaded nanoemulsion with Aloe vera gel in diabetic rats. J. Drug Deliv. Sci. Technol..

[36] Kudłacik-Kramarczyk S., Drabczyk A., Głąb M., Alves-Lima D., Lin H., Douglas T., Kuciel S., Zagórska A., Tyliszczak B. (2021). Investigations on the impact of the introduction of the Aloe vera into the hydrogel matrix on cytotoxic and hydrophilic properties of these systems considered as potential wound dressings. Mater. Sci. Eng. C.

[37] Praseetha P.K., Vibala B.V., Sreedevy K., Vijayakumar S. (2021). Aloe-vera conjugated natural Carbon Quantum dots as Bio-enhancers to accelerate the repair of chronic wounds. Ind. Crop. Prod..

[38] Akhdar M., Abedini R., Tavakolpour S., Gholibeigian Z., Azizpour A. (2021). A randomized, double-blind, placebo-controlled trial of a commercial Aloe vera gel for mitigation of phototherapy side-effects in vitiligo patients. J. Herb. Med..

[39] Sharifi E., Chehelgerdi M., Fatahian-Kelishadrokhi A., Yazdani-Nafchi F., Ashrafi-Dehkordi K. (2021). Comparison of therapeutic effects of encapsulated Mesenchymal stem cells in Aloe vera gel and Chitosan-based gel in healing of grade-II burn injuries. Regen. Ther..

[40] Poordast T., Ghaedian L., Ghaedian L., Najib F.S., Alipour S., Hosseinzadeh M., Vardanjani H.M., Salehi A., Hosseinimehr S.J. (2020). Aloe Vera; A new treatment for atrophic vaginitis, A randomized double-blinded controlled trial. J. Ethnopharmacol..

[41] Cavasana A.L., dos Santos C.H.M., Dourado D.M., Guimarães F.D.S., Barros F.H.R., de Campos G.C.O., Leme G.A.L., da Silva L.D.M., Wahl L.M., Gutterres N.B.D.A. (2019). Effectiveness of the Aloe Vera extract in the treatment of fistula-in-ano. J. Coloproctol..

[42] Paul K., Darzi S., Del Borgo M.P., Cousins F.L., Werkmeister J.A., Gargett C.E., Mukherjee S. (2020). Vaginal delivery of tissue engineered endometrial mesenchymal stem/stromal cells in an aloe vera-alginate hydrogel alleviates maternal simulated birth injury. Appl. Mater. Today.

[43] Alamolhoda S.H., Mirabi P., Mojab F. (2020). Effects of both Aloe Vera gel and breast milk on the improvement of nipple soreness in lactating women—A randomized controlled trial. J. Herb. Med..

[44] Gharaboghaz M.N.Z., Farahpour M.R., Saghaie S. (2020). Topical co-administration of Teucrium polium hydroethanolic extract and Aloe vera gel triggered wound healing by accelerating cell proliferation in diabetic mouse model. Biomed. Pharmacother..

[45] Guleken Z., Depciuch J., Ege H., Ilbay G., Kalkandelen C., Ozbeyli D., Bulut H., Sener G., Tarhan N., Kuruca S.E. (2021). Spectrochemical and biochemical assay comparison study of the healing effect of the Aloe vera and Hypericum perforatum loaded nanofiber dressings on diabetic wound. Spectrochim. Acta Part A Mol. Biomol. Spectrosc..

[46] Liebert M.A. (1984). Final report on the safety assessment of methylparaben, ethylparaben, propylparaben, and butylparaben. J. Am. Coll. Toxicol..

[47] Nowak K., Jabłońska E., Ratajczak-Wrona W. (2020). Controversy around parabens: Alternative strategies for preservative use in cosmetics and personal care products. Environ. Res..

[48] Howard K. Reviewed by Rachel Nazarian, MD, FAAD Board-Certified Dermatologist on 24 July 2021. Carbomers Are in Thousands of Skincare Products—Here’s What They Do by. https://www.byrdie.com/carbomer-4777780.

[49] Purnamawati S., Indrastuti N., Danarti R., Saefudin T. (2017). The Role of Moisturizers in Addressing Various Kinds of Dermatitis: A Review. Clin. Med. Res..

[50] National Center for Biotechnology Information PubChem Compound Summary for CID 10442, 1,3-Propanediol. Updated 23 January 2021. https://pubchem.ncbi.nlm.nih.gov/compound/1_3-Propanediol.

[51] Shunatona B. Reviewed by D. Engelman Board-Certified Dermatologist on 29 June 2020. What You Should Know about Propanediol in Your Skincare Products. A Complete Guide. https://www.byrdie.com/propanediol-for-skin-4773491#citation-2.

[52] Shunatona B. Reviewed by R. Nazarian, MD, FAAD Board-Certified Dermatologist on 27 June 2020. If You’ve Never Heard of Triethanolamine, Here’s What to Know. A Dermatologist and Cosmetic Chemist Explain. https://www.byrdie.com/triethanolamine-for-skin-4777052#citation-1.

[53] Fiume M.M., Heldreth B., Bergfeld W.F., Belsito D.V., Hill R.A., Klaassen C.D., Liebler D.C., Marks J.G., Shank R.C., Slaga T.J. (2017). Safety Assessment of Diethanolamine and Its Salts as Used in Cosmetics. Int. J. Toxicol..

[54] Derseg Co. Casos de Éxito. http://www.derseg.com/exito.php.

[55] Tüysüz H., Schüth F. (2012). Ordered Mesoporous Materials as Catalysts. Adv. Catal..

[56] Tovar-Carrillo K.L., Sueyoshi S.S., Tagaya M., Kobayashi T. (2013). Fibroblast Compatibility on Scaffold Hydrogels Prepared from Agave Tequilana Weber Bagasse for Tissue Regeneration. Ind. Eng. Chem. Res..

[57] Ali W., Gebert B., Altinpinar S., Mayer-Gall T., Ulbricht M., Gutmann J.S., Graf K. (2018). On the Potential of Using Dual-Function Hydrogels for Brackish Water Desalination. Polymers.

[58] Dorsett-Martin W.A. (2004). Rat models of skin wound healing: A review. Wound Repair Regen..

[59] Garros I.D.C., Campos A.C.L., Tambara E.M., Tenorio S.B., Torres J.M., Arruda D.M. (2006). Extrato de passiflora edulis na cicatrização de feridas cutâneas abertas em ratos: Estudo morfológico e histológico. Acta Cir. Bras..

[60] González Tuero J. (2014). Efecto del limo de la salina de Guantánamo en la cicatrización por segunda intención en ratas. MediSan.

[61] Giusto G., Vercelli C., Comino F., Caramello V., Tursi M., Gandini M. (2017). A new, easy-to-make pectin-honey hydrogel enhances wound healing in rats. BMC Complement. Altern. Med..

[62] Otálora M., Wilches-Torres A., Castaño J. (2021). Extraction and Physicochemical Characterization of Dried Powder Mucilage from *Opuntia ficus-indica* Cladodes and Aloe Vera Leaves: A Comparative Study. Polymers.

[63] Bendjelloul M., Elandaloussi E.H., De Ménorval L.-C., Bentouami A. (2016). Quaternized triethanolamine-sebacoyl moieties in highly branched polymer architecture as a host for the entrapment of acid dyes in aqueous solutions. J. Water Reuse Desalin..

[64] Tamada Y., Ikada Y. (1993). Effect of Preadsorbed Proteins on Cell Adhesion to Polymer Surfaces. J. Colloid Interface Sci..

